# Proper application of anticoagulation therapy on cancer-associated venous thrombosis

**DOI:** 10.1007/s44313-024-00029-3

**Published:** 2024-08-02

**Authors:** Ho-Young Yhim

**Affiliations:** Department of Internal Medicine, Jeonbuk National University Medical School, 20 Geonji-Ro, Deokjin-Gu, Jeonju, 54907 Republic of Korea

**Keywords:** Anticoagulation, Cancer, Venous thromboembolism

## Abstract

Cancer-associated venous thromboembolism (VTE) significantly impacts morbidity and mortality. The introduction of direct oral anticoagulants over the past decade has revolutionized VTE treatment in patients with active cancer, offering potential advantages over traditional therapies. However, uncertainties persist regarding the optimal selection and dosage of anticoagulants, particularly in patients with specific risk factors for bleeding, such as certain cancer types (e.g., upper gastrointestinal cancer, genitourinary cancer, primary or metastatic brain tumor, and hematologic malignancies) and specific patient characteristics (e.g., renal dysfunction and thrombocytopenia). Recent data on the thrombotic risk associated with low thrombotic burden VTE, such as subsegmental pulmonary embolism and isolated distal deep vein thrombosis, underscore the need for updated management strategies in daily clinical practice. This review aims to explore these issues and highlight the evolving landscape of cancer-associated VTE management.

## Introduction

Venous thromboembolism (VTE), including pulmonary embolism (PE) and lower extremity deep vein thrombosis (DVT), is a relatively common complication in patients with cancer, accounting for approximately 20% of all VTE cases [1–4]. Cancer-associated VTE contributes significantly to morbidity and mortality, being the second leading cause of death among patients with cancer after cancer progression [5]. Besides mortality, it is associated with a significant psychological burden, an increase in hospitalization, and the potential for interruptions and delays in ongoing cancer treatments [6, 7]. The need for appropriate anticoagulation therapy in cancer-associated VTE patients has been well established for decades. Recently, several large-scale prospective randomized trials have demonstrated the efficacy of direct oral anticoagulants (DOACs) in treating patients with cancer-associated VTE [8–13]. However, challenging issues remain in the diagnosis and management of cancer-associated VTE, such as the management of asymptomatic low thrombotic burden VTE and the optimal dose and duration of DOACs for extended therapy. Therefore, this review aims to examine treatment strategies for cancer-associated VTE based on the latest evidence and evolving drugs.

### Treatment of low thrombotic burden VTE in patients with cancer

The decision regarding anticoagulation therapy in patients with cancer and symptomatic acute proximal DVT and/or PE is relatively straightforward, with the majority of international guidelines recommending anticoagulation therapy unless there is a high risk for bleeding [14–18]. However, advancements in diagnostic imaging techniques and the widespread use of contrast-enhanced imaging to evaluate disease status in patients with cancer have led to increased detection of VTE, even when symptoms are minimal or absent [19]. In fact, approximately 40–50% of PE in patients with cancer are incidentally detected [20–22]. Despite being incidentally diagnosed, current standard treatment recommendations advocate for anticoagulation therapy for incidental PE [14–17], as data suggest that the risk of recurrent VTE is not different from symptomatic VTE [21, 23]. Nevertheless, controversy remains regarding the treatment of incidental VTE with low thrombotic burden, such as subsegmental PE and lower extremity distal DVT. Given the lack of evidence that VTE treatment in patients with cancer improves disease control and survival, one of the primary goals of cancer-associated VTE treatment is eventually to prevent fatal thromboembolic events. This raises the question of whether asymptomatic low thrombotic burden patients should receive the same management strategy as other cancer-associated VTE treatments.

A retrospective study investigated the clinical outcomes of 93 patients with isolated subsegmental PE who did not have DVT [24]. The study analyzed recurrence, hemorrhage, and mortality at three months based on whether anticoagulation therapy was administered. Among the 71 patients who received anticoagulation therapy, one experienced VTE recurrence, eight experienced bleeding (5 major, 3 minor), and two experienced non-VTE-related deaths. In contrast, among the 22 patients who did not receive anticoagulation therapy, there were no occurrences of VTE recurrence, hemorrhage, or death at three months. These findings suggest that anticoagulation therapy could be deferred in patients with isolated subsegmental PE who had sufficient cardiopulmonary reserve and no thrombus in the lower extremities, and re-evaluation could be performed after one to two weeks [25, 26]. However, this study included a very small number of patients with cancer-associated subsegmental PE. Consequently, it remains uncertain whether this conservative strategy can be applied to the management of subsegmental PE in patients with cancer.

An individual patients-level data meta-analysis conducted by van der Hulle et al*.* [27] compared recurrence, bleeding, and mortality between incidental subsegmental PE and more proximal PE in patients with cancer. The study demonstrated that the clinical outcomes of cancer-associated isolated subsegmental PE might differ from those not associated with cancer. The study included 923 patients with cancer-associated incidental PE, 193 of whom had isolated incidental subsegmental PE. It found that the 6-month recurrence rate, major bleeding, and overall mortality in patients with subsegmental PE did not differ from those with more proximal PE. Moreover, a recent international prospective cohort study on cancer-associated incidental PE indicated that the risk of recurrent VTE in subsegmental PE was comparable to that in more proximal PE, suggesting that therapeutic anticoagulation is recommended for cancer-associated isolated subsegmental PE [28].

A recent report on the management of isolated distal DVT, defined as thrombosis confined to the infrapopliteal veins of the lower extremities, provides insight into another form of low thrombotic burden VTE. Galanaud et al*.* [29] used data from the international Registro Informatizado de la Enfermedad Tromboembolica venosa (RIETE) registry to compare the risk of VTE recurrence, major bleeding, and death in cancer-associated distal DVT with those in cancer-associated proximal DVT and non-cancer-associated distal DVT. They found that the risk of VTE recurrence, major bleeding, and death in cancer-associated distal DVT was significantly higher than that in non-cancer-associated distal DVT. Notably, the risks of VTE recurrence and death in cancer-associated distal DVT were similar to those in cancer-associated proximal DVT. Thus, although distal DVT may have a low thrombotic burden, the data support the administration of anticoagulation therapy in patients with cancer-associated distal DVT. Hence, current evidence indicates that the risk of recurrent VTE in patients with incidental subsegmental PE or isolated distal DVT is not negligible, warranting therapeutic anticoagulation unless there is a high risk of bleeding.

### Optimal choice and dosage of anticoagulant in cancer-associated VTE

Since the early 2000s, low molecular weight heparin (LMWH) has been the standard of care for the treatment of cancer-associated VTE. However, significant advances in anticoagulation therapy have occurred over the past 7–8 years. DOACs, which primarily inhibit clotting factors such as factor Xa and thrombin through oral administration, offer several advantages over LMWH and other anticoagulants, including oral dosing, fixed dosing without the need for laboratory monitoring, and the absence of drug-food interactions. Moreover, prospective randomized trials [8–13] and meta-analyses [30] comparing the efficacy and safety of LMWH and DOACs in cancer-associated VTE have shown that DOACs reduce the risk of recurrent VTE by approximately 30%. While there is variability in the risk of major bleeding between studies, a significant increase in clinically relevant non-major (CRNM) bleeding has been associated with DOACs [30]. Based on these data, international guidelines on the management of VTE in most patients with cancer recommend DOACs as the anticoagulant of choice [14–18]. However, despite DOACs becoming the standard of care for cancer-associated VTE treatment, there remain unmet clinical needs in anticoagulation therapy. Specifically, in cases where the risk of bleeding increases with the use of DOACs, such as in certain cancer types (e.g., upper gastrointestinal and genitourinary cancers) [8, 9, 31] and due to drug-to-drug interactions with medications affecting cytochrome 3A4 and p-glycoprotein [32, 33], LMWH is recommended over DOACs [18]. Moreover, all clinical trials comparing DOACs with LMWH have excluded patients with severe renal dysfunction (CrCl < 30 mL/min), resulting in insufficient evidence for the use of DOACs in such cases. Similarly, there is a lack of evidence supporting to use of DOACs as the standard anticoagulant in VTE treatment for patients with cancer and CNS metastasis or hematologic malignancies [34]. Thus, while DOACs are considered the anticoagulant of choice in most hemodynamically stable cancer-associated VTE, future research should focus on finding appropriate treatments for certain high-risk cancer types (e.g., upper gastrointestinal cancer, genitourinary cancer, primary or metastatic brain tumor, and hematologic malignancies) and specific patient characteristics (e.g., renal dysfunction and thrombocytopenia).

In this context, there is growing interest in anticoagulants targeting factor Xl, a crucial component of the contact coagulation pathway. Factor XI has been identified as an attractive target for anticoagulants because it can mitigate thrombosis without impacting hemostasis [35, 36]. Abelacimab, a monoclonal antibody targeting factor XI, has garnered attention for its potential role in treating cancer-associated VTE. This interest is driven by its long half-life, allowing for monthly dosing, and its administration via parenteral injection, which is independent of gastrointestinal absorption [37, 38]. Two large randomized phase 3 trials, ASTER (NCT05171049) and MAGNOLIA (NCT05171075), are currently underway to compare the efficacy and safety of abelacimab with DOACs or LMWH.

### Optimal duration of anticoagulation therapy in patients with cancer and VTE

In patients with cancer-associated VTE, the decision to extend anticoagulation therapy beyond 6 months primarily depends on balancing the risks and benefits related to thrombosis recurrence and bleeding complications. The risk of VTE recurrence in patients with cancer persists as long as active cancer is present; hence, the current standard of care recommends extended anticoagulation therapy if the risk of bleeding is not high [14–17]. However, the administration of anticoagulants carries an inevitable cumulative risk of major bleeding events, approximately at a rate of 0.7% per month during the extended period beyond 6 months [39]. In this context, the EVE trial was conducted to compare reduced-dose apixaban with standard therapy using full-dose apixaban during the extended treatment period. The aim was to assess whether the risk of bleeding could be reduced without compromising the efficacy of anticoagulation [40]. The EVE trial compared reduced-dose apixaban (2.5 mg twice daily) with full-dose apixaban (5 mg twice daily) in 370 patients with cancer-associated VTE who received 6 to 12 months of anticoagulation therapy at the time of study enrollment. The primary endpoint of the study, comprising major bleeding plus CRNM bleeding, did not reveal a statistically significant difference between the two groups. However, the reduced-dose group exhibited a numerically lower rate (8.9% vs. 12.2%, p = 0.39). Furthermore, there was no significant difference in VTE recurrence between the reduced-dose and full-dose apixaban groups (5.0% vs. 4.4%, p = 1.0). These findings suggest that apixaban 2.5 mg twice daily could be applicable for extended anticoagulation therapy in patients with active cancer and VTE. However, further results from the ongoing API-CAT trial (NCT03692065), which involves a larger cohort (n = 1,722), are awaited for confirmation. Therefore, until more substantial evidence is available for the use of reduced-dose DOACs, full-dose DOACs are still recommended for patients with active cancer even beyond 6 months of anticoagulation if the risk of bleeding is not high, and frequent re-evaluation of the risk/benefit of anticoagulation therapy is necessary. However, based on the recent report from the EVE trial involving reduced-dose apixaban, reduced-dose DOACS may emerge as another viable treatment option for extended anticoagulation in the future.

## Conclusion

In conclusion, the introduction of DOACs over a decade ago revolutionized the management of VTE, establishing DOACs as the standard of care for most patients with cancer-associated VTE. However, despite this advancement, the landscape of VTE treatment continues to evolve, and a universal treatment approach is not yet realistic. Therefore, further research is essential to optimize the use of DOACs in the treatment of cancer-associated VTE. This includes identifying the optimal choice of anticoagulant, determining the most optimal drug dosage, and establishing the ideal duration of treatment.

## Data Availability

No datasets were generated or analysed during the current study.
